# Defining Activity Thresholds Triggering a “Stand Hour” for Apple Watch Users: Cross-Sectional Study

**DOI:** 10.2196/53806

**Published:** 2024-06-10

**Authors:** Katy Lyons, Alison Hau Hei Man, David Booth, Graham Rena

**Affiliations:** 1 Division of Cellular and Systems Medicine Ninewells Hospital and Medical School University of Dundee Dundee United Kingdom; 2 School of Life Sciences University of Dundee Dundee United Kingdom

**Keywords:** stand hour, Apple Watch, sedentary behavior, light physical activity, cardiovascular disease, type 2 diabetes, data collection, wearable, wearables, watch, smartwatch, stand, standing, sedentary, physical activity, exercise, movement, algorithm, algorithms, predict, predictive, predictor, predictors, prediction, machine learning

## Abstract

**Background:**

Sedentary behavior (SB) is one of the largest contributing factors increasing the risk of developing noncommunicable diseases, including cardiovascular disease and type 2 diabetes. Guidelines from the World Health Organization for physical activity suggest the substitution of SB with light physical activity. The Apple Watch contains a health metric known as the stand hour (SH). The SH is intended to record standing with movement for at least 1 minute per hour; however, the activity measured during the determination of the SH is unclear.

**Objective:**

In this cross-sectional study, we analyzed the algorithm used to determine time spent standing per hour. To do this, we investigated activity measurements also recorded on Apple Watches that influence the recording of an SH. We also aimed to estimate the values of any significant SH predictors in the recording of a SH.

**Methods:**

The cross-sectional study used anonymized data obtained in August 2022 from 20 healthy individuals gathered via convenience sampling. Apple Watch data were extracted from the Apple Health app through the use of a third-party app. Appropriate statistical models were fitted to analyze SH predictors.

**Results:**

Our findings show that active energy (AE) and step count (SC) measurements influence the recording of an SH. Comparing when an SH is recorded with when an SH is not recorded, we found a significant difference in the mean and median AE and SC. Above a threshold of 97.5 steps or 100 kJ of energy, it became much more likely that an SH would be recorded when each predictor was analyzed as a separate entity.

**Conclusions:**

The findings of this study reveal the pivotal role of AE and SC measurements in the algorithm underlying the SH recording; however, our findings also suggest that a recording of an SH is influenced by more than one factor. Irrespective of the internal validity of the SH metric, it is representative of light physical activity and might, therefore, have use in encouraging individuals through various means, for example, notifications, to reduce their levels of SB.

## Introduction

Sedentary behavior (SB) is a prevalent factor contributing to all-cause mortality and increasing the risk of developing noncommunicable diseases [1]. SB has been becoming more common for decades, and this has been exacerbated by the recent pandemic. After the pandemic, 47% of the British workforce worked from home [2]; the amount of SB in university students increased by 3 hours per day [3], and the pandemic is likely to have brought further isolation to the most sedentary segment of older adults, who can spend upwards of 8.5 hours a day sitting and who are also most vulnerable to developing noncommunicable diseases [4].

The detrimental physiological effect of SB on risk factors for noncommunicable diseases such as cardiovascular disease (CVD) and type 2 diabetes (T2D) may manifest independently of levels of moderate to vigorous physical activity [5]. Guidelines from the World Health Organization (WHO) for physical activity suggest the substitution of SB with light physical activity (LPA) but do not recommend a guideline on the amount of LPA that should be undertaken, focusing instead on guidelines for moderate to vigorous physical activity [6].

There is a paucity of evidence on the effect LPA has on reducing the risk of developing CVD and T2D; however, studies have been carried out to investigate the effects of LPA on known CVD risk factors, including hypertension [7] and resting heart rate (RHR). The addition of LPA to break up SB lowers both systolic and diastolic blood pressure compared with those who remain sedentary [8]. It has been suggested that the magnitude of effects observed could reduce the risk of coronary heart disease by around 4%-5% [8]. Higher RHR independently associated with increased CVD mortality in apparently healthy people and in those with coronary heart disease [9,10]. Higher levels of daily standing have been found to inversely correlate with RHR [11]. In addition, exercise has been found to reduce atherogenic markers such as non–high-density lipoprotein cholesterol and apolipoprotein B, which are responsible for the build-up of plaque and atheroma formation [12]. With regards to T2D, short and regular periods of LPA integrated into sedentary periods every 20 minutes reduce the postprandial levels of glucose [13]. This effect was seen in both healthy individuals and individuals already diagnosed with T2D. LPA also has beneficial physiological effects on the ability of muscles to uptake plasma glucose and insulin sensitivity [12,14].

Previous studies investigating breaks in SB have often used accelerometry to measure physical activity levels [15,16]. With the advancement of technology, many wrist-worn health and fitness trackers now have the means to measure accelerometry, amongst other activity measures. They are more frequently worn in everyday life, giving a nonintrusive insight into the consequences of physical activity levels in the community.

This study focuses on the Apple Watch metric known as the stand hour (SH). The stand goal on these watches is completed by standing and moving around for at least 1 minute every hour for a target minimum of 12 different hours in the day. The SH is intended to provide motivation and encouragement to decrease SB by promoting stand intervals every hour. It is undetermined whether the Apple SH has any physiological impact on decreasing the risk of developing noncommunicable diseases.

This cross-sectional study aimed to use the cross-sectional data collected from free-living participants, to identify other watch measurements that may contribute to creating an SH recording. This will enable us to understand what physiological measures the Apple Watch uses to determine whether an individual has stood up and moved each hour. Furthermore, this study plans to estimate the value of any significant SH predictors that deem that an individual has stood, that is, the threshold at which the metric turns from 0 to 1.

## Methods

### Ethical Considerations

Ethical approval to undertake this project was given by the University of Dundee School of Medicine Research Ethics Committee (20/55) prior to participant recruitment and data collection.

### Exclusion Criteria and Recruitment

Convenience sampling occurred via social media, word of mouth, and email dispersal to recruit individuals throughout the United Kingdom. Involvement was completely voluntary, granting participants permission to withdraw themselves from the study at any point. Individuals’ participation in the study was excluded if individuals were outside the age range of 18 to 60 years, had long-term health conditions, took prescribed medication, were receiving medical treatment, or lived outside the United Kingdom. A Strengthening the Reporting of Observational Studies in Epidemiology (STROBE) checklist was completed during the preparation of this paper.

### Data Collection

Participants were required to fill out a consent form and a preanonymized Personal Health Questionnaire (PHQ). Upon completion, they downloaded a third-party app, Health Auto Export to CSV. Through this app, the participants were able to sync data collected on their watch from the health app on their iPhone and export the data in the form of an Excel (Microsoft) file. The Excel file was then anonymized by the participant by renaming the file with the corresponding ID number found on their PHQ. All 3 documents were then uploaded onto a secure One Drive (Microsoft) folder. Each participant gave a month’s worth of data in hourly increments from a standardized time period (August 1-31, 2022). Choosing a time period in the past, before participants were recruited, allowed for the observation of free-living individuals unperturbed by observer bias. The study had 20 participants; this number is based on a previous study we had carried out [11].

Active energy (AE; kJ), average heart rate (HR [avg]; count per minute), step count (SC; count), and walking and running distance (WRD; km) were selected to investigate their effect on completing an SH. These 4 variables represent a range of physiological measurements, relating to the effect of activity (SC and WRD) and normal body measurements (HR and AE). HR (avg) was chosen as it was the only HR measurement that gave an indication of HR throughout the whole hour.

This study only considered Apple Watch–derived measurements. The iPhone health app automatically measures SC and WRD if an iPhone is present on a person’s body. Therefore, the data collected included iPhone measurements, as well as Apple Watch measurements. Watch measurements were distinguished from iPhone measurements as HR is continually recorded on a watch, but the iPhone has no technology to measure HR. Any hourly measurements that had absent HR data were removed from the data set. A total of 1901 (17%) data points were excluded from the study due to this.

### Measurements

#### Active Energy

AE records how much extra energy the body has consumed through exercise from the base level of resting energy expenditure, measured in kJ. The accuracy and reliability of this measurement have been studied in watches from several manufacturers and seem to be poor [17,18].

#### Stand Hour

On Apple smartwatches, an SH measurement is intended to be recorded by performing at least 1 minute of movement within an hour. However, there is no further guidance provided by Apple regarding the quantity or type of movement needed to achieve this. It is thought that Apple watches use an accelerometer and gyroscope to identify standing and movement [19]. A previous study using monthly data from one smartwatch noted that an SH was recorded 100% (n=177) of the time if 251 steps per hour have been taken, yet 8% (n=251) of the time an SH was recorded, 0 steps per hour were taken [11].

#### Heart Rate

The accuracy and precision of HR measurements have been trialed against ECG monitors, as well as other leading technologies on the market including Polar branded chest straps, forearm monitors, and other brand-leading, HR-measuring, wrist-worn devices such as Fitbit (Google) and Garmin (Garmin Ltd). Apple watches outperformed all other wrist-worn devices and forearm monitors providing a Lin concordance coefficient value of 0.92 agreement to the ECG [20].

#### Step Count

SC is measured using an accelerometer. SC on Apple watches has been validated against video recordings and hand-counted measurements. The Apple Watch recorded a Lin concordance coefficient value of 0.96, showing an extremely strong correlation between the 2 methods [21].

#### Walking and Running Distance

Distance walked or run was recorded every hour in kilometers (km). This measurement is recorded by the use of GPS. A previous study found no statistically significant differences between GPS distance measurements on the Apple Watch series 4, compared with a premeasured distance, measured by a trundle wheel [22].

### Statistical and Data Analysis

Statistical analyses were performed and related graphs were created on R Studio (R Core Team) or Prism (GraphPad). Maximum and minimum adequate models were fitted as a generalized linear model with mixed effects (GLMM). The fixed effect model from these GLMMs report estimate (SE), followed by a *P* value and significance marking. The random effects model reports variance with SD, and a stepwise backward regression was performed to create the minimum adequate model from the maximal model. ANOVA test using type II Wald *χ*^2^ test was used to confirm the findings of the GLMM [23].

Means and medians of the significant variables were calculated and graphically visualized on either bar charts or box plots and the differences were statistically tested for significance using Student *t* tests (mean) and Wilcoxon summed ranked tests (median) [24].

To investigate and understand the threshold at which the variables change the SH metric from 0 to 1, a graphical visualization of the fitted SH was created using marginal estimated means calculated from the data set for each variable to investigate them as separate entities. The values obtained were then applied back into the data set to see how accurate the estimated values were.

## Results

### Data From Participant Cohort

Twenty individuals participated in this study, with a total of 9236 hourly observations between participants. A total of 7301 recorded SHs and 1936 nonrecorded SHs were observed. Cohort demographics are shown in Table 1.

**Table 1:** Study cohort demographic based upon the information gathered from completed PHQ^a,b^.

Demographics			Participants, n (%)
**Sex**			
	Male	4 (20)	
	Female	16 (80)	
**Age (years)**			
	18-29	13 (65)	
	30-39	2 (10)	
	40-49	2 (10)	
	50-60	3 (15)	
**BMI (kg/m^2^)**			
	<20	1 (5)	
	20-24.99	8 (40)	
	25-29.99	8 (40)	
	30-34.99	1 (5)	
	Unknown	2 (10)	
**Apple Watch model**			
	Series 3	2 (10)	
	Series 4	1 (5)	
	Series 5	4 (20)	
	Series 6	5 (25)	
	Series 7	2 (5)	
	Series SE	6 (30)	

^a^The total number of participants that were included in this study was 20, 16 of which were female and 4 male. The age range was collected in groups of 10 years, with the youngest group within the 18-29–year bracket, and the oldest in the 50-60–year age bracket. The largest majority of participants in the study were females aged 18-29 years. BMI levels varied within the cohort, ranging between <20 kg/m^2^ and 34.99 kg/m^2^. These categories of BMI were deemed “underweight” (<20 kg/m^2^), “healthy” (20.00 kg/m^2^-24.99 kg/m^2^), “overweight” (25.00 kg/m^2^-29.99 kg/m^2^), and “obese” (30 kg/m^2^-34.99 kg/m^2^). Similarly, this study contained data collected over a range of older and newer models of the Apple Watch spanning from series 3 to series 7. Each result is recorded as a number (n) and its respective percentage value (%).

^b^PHQ: Personal Health Questionnaire.

### Exploring the Measurements Contributing to an SH Recording

Tables 2 and 3 show the output from the most maximum and minimum adequate model fitted as a GLMM. As shown in Table 2, the maximum model was built considering all the fixed measurements that were chosen for further investigation. AE, SC, WRD, and HR (avg) were the measurements recorded on the watch while gender and age were the factors obtained from the PHQ. The minimum model removed any insignificant measurements from the SH. Table 2 shows that both AE (0.021, SE 0.002) and SC (0.014, SE 0.004) produced statistically significant coefficient estimates (*P*<.05). However, WRD (7.837, SE 5.46) did not have a significant *P* value (*P*=.15). Table 3 reports the random effects of the model and the variance shows the spread of the data between each participant. The value of variance increased in the minimum adequate model, from 0.194 to 0.245.

**Table 2:** The fitted model from GLMM^a^ output^b^.

		Maximum model		Minimum adequate model	
		Estimate (SE)	*P* value	Estimate (SE)	*P* value
Intercept		–3.146 (0.381)	<.001^c^	–2.710 (0.151)	<.001^c^
AE^d^		0.020 (0.002)	<.001^c^	0.021 (0.002)	<.001^c^
SC^e^		0.014 (0.003)	<.001^c^	0.014 (0.004)	.001^c^
WRD^f^		7.848 (3.710)	.03^g^	7.837 (5.46)	.15
HR (avg)^h^		0.005 (0.005)	.34	N/A^i^	N/A
Gender (male)		–0.059 (0.296)	.84	N/A	N/A
**Age (years)**					
	30-39	0.774 (0.390)	.05	N/A	N/A
	40-49	–0.023 (0.390)	.95	N/A	N/A
	50-60	0.341 (0.322)	.30	N/A	N/A

^a^GLMM: generalized linear model with mixed effects.

^b^A generalized linear model was designed to assess which watch-recorded variables play a role in influencing the recording of a stand hour (SH). The output from the generalized linear model is shown. Both the maximum model and the most simplified minimum adequate model, generated by performing a stepwise backward regression on R studio are shown. The intercept denotes the likelihood an SH was recorded by the most average participant in the study before the addition of any influential factors. The model reports the estimate and SE for each variable included in the model. Only significant factors (*P*<.05) present in the maximum model were present in the minimum adequate model—active energy and step count. Values for estimate and SE have been rounded to 3 decimal places where appropriate.

^c^*P*<.05.

^d^AE: active energy.

^e^SC: step count.

^f^WRD: walking and running distance.

^g^*P*<.01.

^h^HR (avg): average heart rate.

^i^N/A: not applicable.

**Table 3:** The random effects of the GLMM, reporting variance, and SD^a^.

Groups		Name	Variance (SD)
**Maximum model^b^**			
	ID	Intercept	0.194 (0.441)
**Minimum adequate model^b^**			
	ID	Intercept	0.245 (0.495)

^a^A generalized linear model was designed to assess which watch-recorded variables play a role in influencing the recording of a stand hour (SH). The output from the generalized linear model is shown. Values for variance and SD have been rounded to 3 decimal places where appropriate.

^b^Number of observations: 9236, groups: ID, 20

The results from Tables 2 and 3 were analyzed further by ANOVA using type II Wald *χ*^2^ test, AE (*W*_1_=169.549; *P*<.001) and SC (*W*_1_=11.639; *P*=.001) and had a statistically significant effect on the recording of an SH. WRD had no statistical significance on the recording in the SH (*W*_1_=2.026; *P*=.15).

Using the coefficient estimates from Table 2, odds ratios were calculated using logit transformations. A logit transformation was applied to the coefficient estimates as the SH metric was restricted to a finite interval (0 or 1) [25]. It is estimated that the Apple Watch recorded one SH 7% of the time when no added factors such as AE or SC were added within the participant cohort. This was calculated using the “intercept” value which is a value representative of the natural activity levels of the most average participant in the study before the addition of SC or AE. In this case, it was a female participant, aged 18-29 years. By increasing the AE value by 1 kJ, the likelihood of an SH being recorded is increased by 2% and for every additional 1 step taken, the likelihood of an SH being recorded increased by 1%.

### Determination of Means and Medians

**Figure 1:**
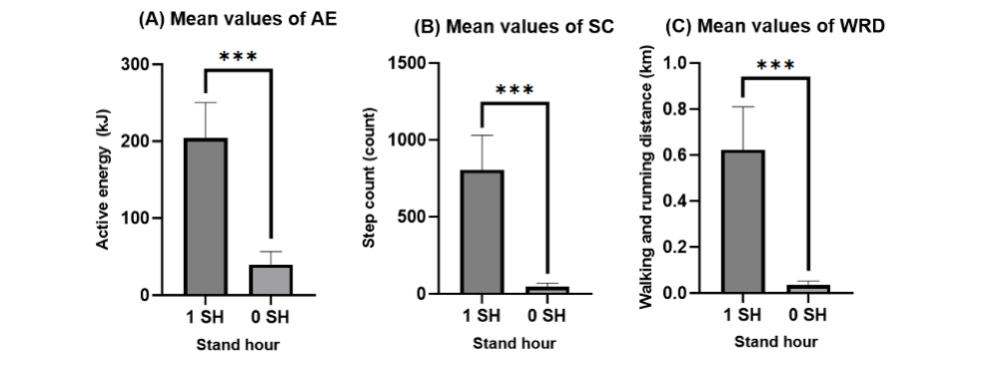
Comparison of the mean AE, SC, and WRD values of 0 and 1-recorded SH. The bar chart shows the mean values with SD. The mean values were calculated between the mean values gathered from each participant in the study cohort (N=20). Graphs were created on Prism. AE: active energy; SC: step count; SH: stand hour; WRD: walking and running distance. ***P<.0001.

Figure 1 shows the mean values of AE, SC, and WRD when an SH was not recorded (0) and when an SH was recorded (1). Student *t* tests were computed to see if there was any significant difference between the mean of recorded SH (1 SH) and non-recorded SH (0 SH) for each measurement. For every measurement, the mean value was statistically different between 0 SH and 1 SH. For AE, the mean value when there was no SH recorded was 39.63 kJ and 204.44 kJ when an SH was recorded (*t*_24.03_=15.05; *P*<.001). For SC, the mean value when there was no SH recorded was 45.62 steps and 803.65 steps when an SH was recorded (*t*_19.42_=14.89; *P*<.001), and for WRD the mean value for WRD when an SH was not recorded was 0.03 km and 0.62 when an SH was recorded (*t*_19.35_=14.02; *P*<.001).

**Figure 2:**
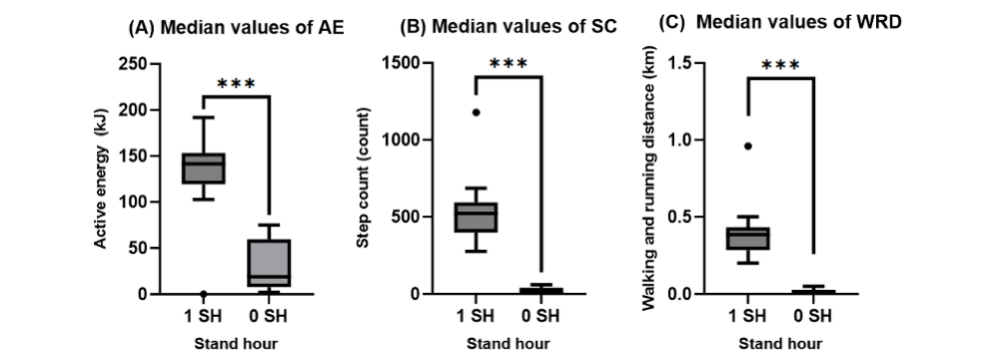
Comparison of the median AE, SC, and WRD values of 0 and 1-recorded SH. Comparing the average median of values when an SH was recorded (1 SH) versus when it was not (0 SH). The median values were calculated between the median value gathered from each participant in the study cohort (N=20). Graphs were created on Prism. AE: active energy; SC: step count; SH: stand hour; WRD: walking and running distance. ***P<.001.

Figure 2 shows the median values of AE, SC, and WRD when an SH was not recorded (0) and when an SH was recorded (1) and confirmed by Wilcoxon rank sum tests to determine the statistical significance of the medians. Again, for all measurements, the median value recorded for 1 SH was significantly higher than those recorded for 0 SH. For AE, the median value when there was an SH was not recorded was 18.96 kJ and 141.47 kJ an SH was recorded (*z*=–4.86; *P*<.001). For SC, the median value when there was no SH recorded was 15.00 steps and 521.59 steps when an SH was recorded (*z*=–5.40; *P*<.001) and for WRD, the median value when there was no SH recorded was 0.01 km and 0.39 km when an SH was recorded (*z*=–5.42; *P*<.001).

### Fitting Models of the SH

**Figure 3:**
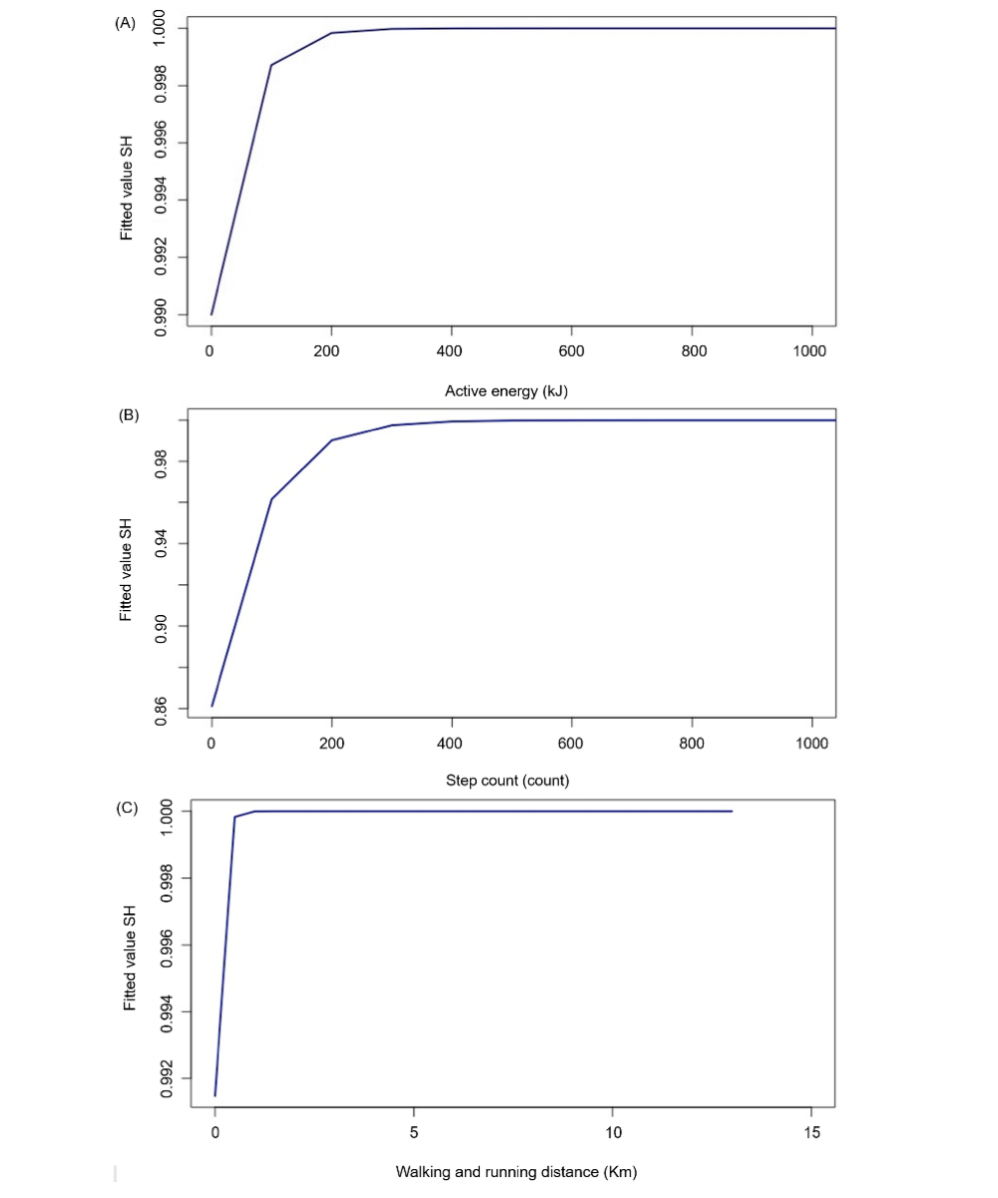
Graphs showing the fitted value of the SH and the impact of increasing the values of AE, SC, and WRD. Analyzing AE, SC, and WRD as separate entities; fitted models were created on R studio using estimated marginal means to predict values where the Apple Watch may record an SH. AE: active energy; SC: step count; SH: stand hour; WRD: walking and running distance.

Figure 3 shows fitted models for the SH, created using estimated marginal means and analyzing each measurement as a separate entity. For each model, values were read at the point where the graph begins to curve sharply, which estimates a threshold above which it becomes much more likely that an SH will be recorded. The turning point where each graph turns sharply indicates where the threshold of values estimates the values are large enough to deem an SH has been sufficiently met. For AE, the graph turns at 0.999 giving an estimated reading of ~100 kJ. For SC, the graph turns at 0.961 to give an estimated value of 97.5 steps and for WRD, the graph turns at 0.999 to estimate a value of 0.468 km. These estimated values were then applied back into the original data set of gathered data to see their accuracy.

A total of 76% (n=5330) of the AE-recorded SH had a value larger than 100 kJ (Figure 3A). It was also shown that 4% (n=7) of the observations recorded as 0 SH had a value larger than 100 kJ. The fitted model for SC (Figure 3B) begins to turn at an earlier point than both the models of AE and WRD, turning at 0.961 rather than 0.999. A total of 92% (n=6682) of the SC-recorded SH had a value larger than 97.5 steps but it also showed that 10% (n=201) of the observations that were recorded as 0 SH had a value larger than 97.5. Only 40% (n=2920) of the WRD-recorded SH observed had a value larger than 0.486 km (Figure 3C). However, it had the least observed 0-recorded SH at 0.05% (n=1).

## Discussion

### Measurements Underpinning the Recording of an SH

AE and SC were found to be significant measurements underpinning the recording of an SH. SC and AE are the most studied measurements across both brand and device types [26]. Apple watch–measured SC has been validated against hip-worn accelerometers in a laboratory-based setting [21] and free-living setting [27]. It is extremely accurate within the laboratory [21] but has been shown to overestimate SC in a free-living setting compared with hip-worn accelerometer [27]. Our finding of the 97.5 SC threshold above which recording of an SH becomes much more likely is consistent with previous evidence showing that 51-100 steps in 1 hour created an SH 95% (n=87) of the time [11]. The AE measurement has been investigated across multiple models of the Apple Watch. Series 6 Apple Watch underestimated the value of energy expenditure at multiple different physical activity intensities including walking (LPA) [17], while the Series 4 Apple Watch also had poor accuracy across multiple exercise intensities [18]. For both SC and AE, there is yet to be a study to compare the values between free-living and laboratory-based environments. It remains inconclusive from the results of our study and from previously documented evidence regarding AE and SC [26,28,29] if there is one measurement that is more effective or has more influence over the other regarding recording an SH. WRD did not prove to be a significant measurement used to record an SH; however, the WRD measurement was included in further analysis as it was the only other measurement that was expressed in the minimum adequate model of the GLMM.

### Changing the SH Metric From 0 to 1

In all three analyzed measurements (AE, SC, and WRD) it was shown that there was a significant difference between both the mean and median values that were needed to create a 1 SH recording and 0 SH recording. From the fitted models, SC proved to be the most accurate within our data set, showing 92% (n=6682) of the recorded SH had a value higher than 97.5 steps. For AE, 76% (n=5330) of the observed 1 SH recorded having a value larger than 100 kJ. The SC value of 97.5 steps is likely to be a feasible and realistic goal to achieve within an hour in many workplaces and other modern lifestyle settings. 100 kJ of consumed energy is 23.90 kcal (calories) [30]. It is estimated that it would take approximately 8-10 minutes of slow-to-moderate paced walking (1.9-3.0 mph) to burn 23.90 kcal [21] with an average male or female body weight (~80 kg and 70 kg, respectively). Given the SH measurement is intended to encourage 1 minute of standing every hour, it is possible that our value of 100 kJ for a change in recording is an overestimate; alternatively, our findings highlight that the SH is not measured by one entity alone, and there is more than one factor influencing the recording of an SH.

### Limitations

Convenience sampling was the most effective method for recruitment due to time constraints. Participants who already owned activity trackers are unlikely to be representative of the whole community. A larger participant cohort more representative of the community would have provided a greater range of natural activity levels, as many of the participants were naturally active. We did not include anybody with CVD, diabetes, or at-risk groups, so the external validity of our findings will need to be established in those groups. Although neither gender, age, nor BMI was found to play a role in affecting the outcome of an SH being recorded, a large majority of our participant cohort was younger females.

### Conclusions

It is currently being investigated whether the detrimental health effects of long periods of SB may be reduced by switching SB for short, regular periods of LPA. This study quantifies the SH metric present within Apple Watch technology. The aim of this metric is that it records standing with movement, for 1 minute in any given hour. The findings of this study reveal the pivotal role of the AE and SC measurements present on the Apple watches, which underlie the algorithm behind the SH recording; however, our findings also suggest that a recording of an SH is influenced by more than one factor. The research from this study finds 100 kJ of energy or 97.5 steps define a threshold for activity above which recording of 1 SH becomes much more likely. Our findings demonstrate that any activity upwards of LPA will trigger an SH to record. Irrespective of the internal validity of the SH metric, the SH is representative of LPA and should, therefore, encourage individuals by any means (including notifications) to promote a reduction in SB by engaging with LPA or greater levels of activity.

## Abbreviations

AE: active energy
CVD: cardiovascular disease
GLMM: generalized linear model with mixed effects
HR: heart rate
HR (avg): average heart rate
LPA: light physical activity
PHQ: Personal Health Questionnaire
RHR: resting heart rate
SB: sedentary behavior
SC: step count
SH: stand hour
STROBE: Strengthening the Reporting of Observational Studies in Epidemiology
T2D: type 2 diabetes
WHO: World Health Organization
WRD: walking and running distance

## Data Availability

The data sets generated and analyzed during this study are available from the corresponding author on reasonable request.
